# Attentional modulation of multisensory integration across the cortical hierarchy

**DOI:** 10.3389/fncir.2026.1920358

**Published:** 2026-08-17

**Authors:** Ye Jin Kim, Seung-Hee Lee

**Affiliations:** 1 Department of Brain and Cognitive Sciences, Korea Advanced Institute of Science and Technology (KAIST), Daejeon, Republic of Korea; 2 Department of Biological Sciences, Korea Advanced Institute of Science and Technology (KAIST), Daejeon, Republic of Korea; 3 Center for Synaptic Brain Dysfunctions, Institute for Basic Science (IBS), Daejeon, Republic of Korea

**Keywords:** association cortex, attention, cortical hierarchy, multisensory integration, prefrontal cortex, sensory cortex

## Abstract

In a multisensory environment, attention prioritizes behaviorally relevant sensory inputs not through a single mechanism but by modulating neural activity across the cortical hierarchy. Yet most studies focus on attentional modulation of unisensory processing and do not specify how attention resolves competition between the senses, or how its effects differ across cortical levels with distinct functional roles. Here, we review human, non-human primate, and rodent studies to argue that attention modulates multisensory processing at three levels. In sensory cortices, it adjusts local response gain and the temporal phase of incoming signals. In association cortices, it shapes the integration of converging inputs, enhancing the attended modality while suppressing competing ones. In the prefrontal cortex, it selects the relevant modality, monitors cross-modal conflict, and resolves the resulting perceptual uncertainty. Critically, these levels do not operate independently. Attention-dependent changes in inter-areal connectivity coordinate modulation across the hierarchy. This review suggests that attentional control of multisensory perception is best understood as a distributed division of labor that extends the priority-map framework of attentional selection to multisensory conditions, in which each cortical level contributes to constructing and updating a unified priority representation of the sensory environment. Attention is therefore an inherently integration-sensitive process, shaped by whether cross-modal signals are integrable, rather than a uniform amplification of sensory signals.

## 1 Introduction

**FIGURE 1:**
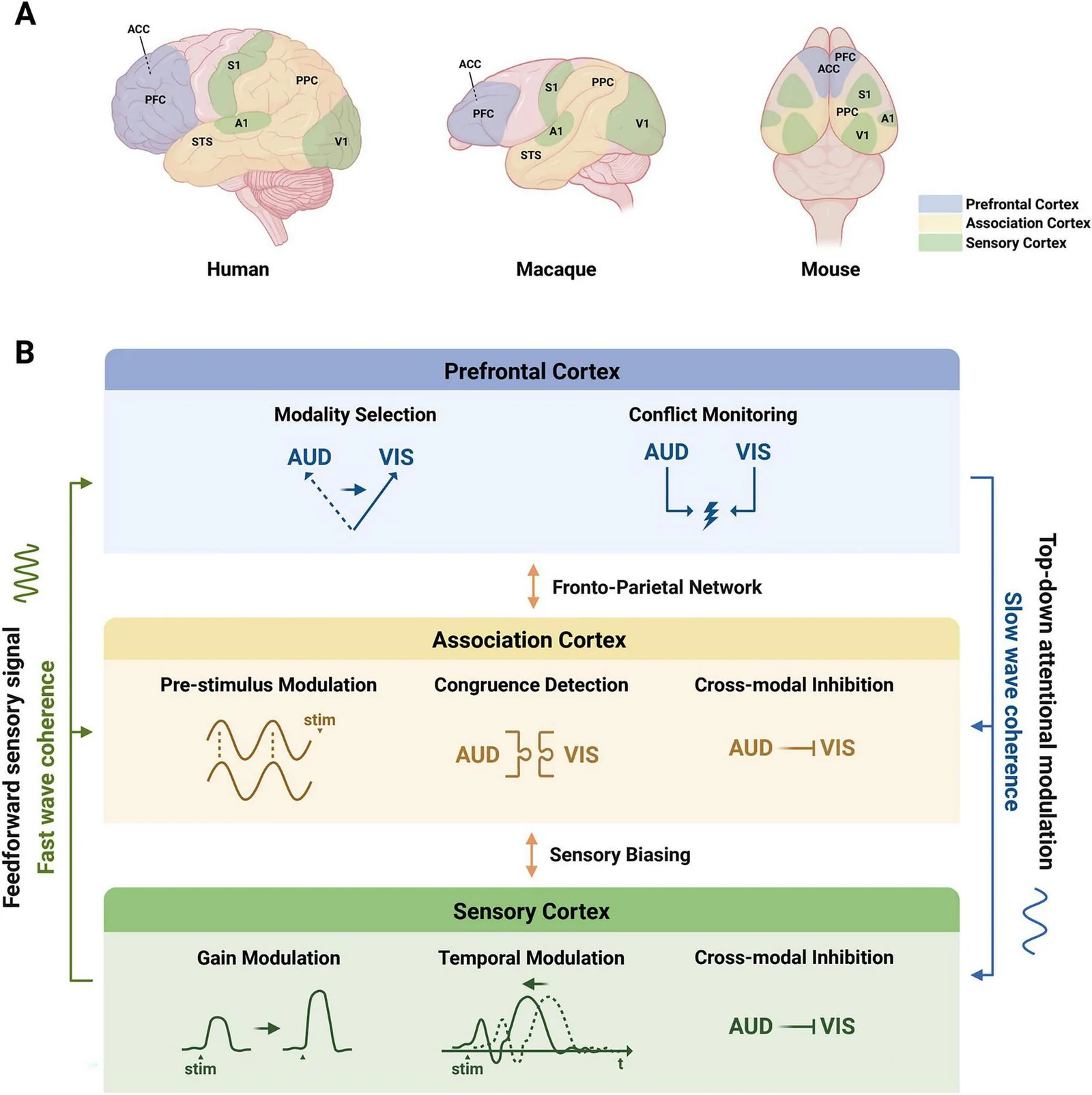
Attentional modulation of multisensory processing across the cortical hierarchy. **(A)** Brain areas involved in attentional modulation of multisensory processing, divided into sensory, association, and prefrontal cortex. Green, sensory cortex; V1, primary visual cortex; A1, primary auditory cortex; S1, primary somatosensory cortex; Yellow, association cortex; PPC, posterior parietal cortex; STS, superior temporal sulcus; Blue, prefrontal cortex; PFC, prefrontal cortex; ACC, anterior cingulate cortex. **(B)** Summary schematic of how multisensory processing across the cortical hierarchy is modulated by attention; color scheme for cortical areas is identical to panel **(A)**.

Attention enables the brain to process relevant sensory information in environments saturated with sensory stimuli from multiple modalities, modulating neural activity to enhance the representation of relevant inputs. This modulation occurs across the cortical hierarchy rather than within a single region (Kastner and Ungerleider, 2000; Reynolds and Heeger, 2009). However, most studies of attentional modulation focus on unisensory processing and do not adequately account for the role of attention in cross-modal integration, and it remains unclear how attention modulates different cortical areas in parallel according to their hierarchical roles during multisensory integration. Therefore, a systematic view of attentional modulation across the cortical hierarchy during multisensory integration is required. In this review, we argue that attentional modulation operates along the cortical hierarchy in distinct ways during multisensory perception. By “hierarchy” we denote a gradient of functional roles rather than a strictly serial feedforward pathway. These levels interact recurrently and through subcortical structures during sensory processing and multisensory integration (Lohse et al., 2026), and such a recurrence across areas is intrinsic to the canonical model of attention, in which working memory, top-down sensitivity control, and competitive selection operate in a recurrent loop (Knudsen, 2007). We first examine how attention modulates local gain, temporal precision, and cross-modal suppression at the level of sensory cortices. Next, we describe how attention modulates the integration process in association cortices. We then review evidence that attention is critical for top-down selection and conflict monitoring in the prefrontal cortex. Finally, we describe how these levels are coordinated through attention-dependent modulation of inter-areal connectivity (Figure 1 and Table 1).

**TABLE 1:** Summary table of the studies listed in this paper.

Modality/task	Sensory cortex			Association cortex		Prefrontal cortex	
	V1/VC	A1/AC	S1	PPC/LIP	STS	LPFC/IFS	ACC
Audiovisual (spatial)	Fairhall and MacAluso, 2009	–	–	Nardo et al., 2014; Bushara et al., 1999; Chambers et al., 2007; Bolognini et al., 2010; Gifford and Cohen, 2004	Fairhall and MacAluso, 2009	Bushara et al., 1999	–
Audiovisual (temporal)	Pervin et al., 2025; Lakatos et al., 2008	Pervin et al., 2025	–	London et al., 2022; Pervin et al., 2025; Yuan et al., 2016	Noesselt et al., 2007	–	–
Audiovisual (illusion)	Mishra et al., 2007, 2010	–	–	Kamke et al., 2012	Keil et al., 2012, 2014	–	–
Audiovisual (speech/semantic conflict)	–	Mayer et al., 2017	–	Mastroberardino et al., 2015	Morís Fernández et al., 2015; Beauchamp et al., 2010	Morís Fernández et al., 2017; Mayer et al., 2017; Noppeney et al., 2010; Gau and Noppeney, 2016; Dong et al., 2024; Mastroberardino et al., 2015	Morís Fernández et al., 2015, 2017; Mayer et al., 2017
Audiovisual (selection / divided attention)	Friese et al., 2016; Gau et al., 2020; Mozolic et al., 2008; Iurilli et al., 2012; Oude Lohuis et al., 2022	Mozolic et al., 2008; Choi and Lee, 2025; Hipp et al., 2011	Iurilli et al., 2012	Shomstein and Yantis, 2004; Su et al., 2020; Frank et al., 2020; Vohn et al., 2007; Song et al., 2017; Choi and Lee, 2025	Friese et al., 2016	Shomstein and Yantis, 2004; Loose et al., 2003; Vohn et al., 2007; Su et al., 2020; Friese et al., 2016; Wimmer et al., 2015; Nakajima et al., 2019	Vohn et al., 2007
Visuo-tactile	Macaluso et al., 2000; Harjunen et al., 2017; Vibell et al., 2007; Weiler et al., 2024	–	Harjunen et al., 2017; Lee et al., 2016	Macaluso et al., 2000	–	–	–
Others (audio-visuo-tactile, olfactory-tactile)	Eimer et al., 2002	Eimer et al., 2002	Eimer et al., 2002	–	–	Birrell and Brown, 2000; Bissonette et al., 2008	–

Studies are classified by brain areas and task types. Review papers are excluded. V1/VC, (primary) visual cortex; A1/AC, (primary) auditory cortex; S1, primary somatosensory cortex; PPC, posterior parietal cortex; LIP, lateral intraparietal cortex; STS, superior temporal sulcus; LPFC, lateral prefrontal cortex; IFS, inferior frontal sulcus; ACC, anterior cingulate cortex.

## 2 Sensory cortex

Sensory cortices perform early processing of sensory information and are well known to be modulated by attention during unisensory processing (Cohen and Maunsell, 2009; Kastner and Ungerleider, 2000; Reynolds and Heeger, 2009). How they are modulated when cross-modal inputs are presented is less well understood. Emerging evidence reveals robust attentional modulation of early sensory cortices during cross-modal integration, including gain control, temporal modulation, and cross-modal inhibition. Because the integrated output of multisensory processing builds on modality-specific representations, modulation at this level directly shapes which signals are preferentially propagated to higher-order areas.

### 2.1 Gain modulation

A prominent form of attentional modulation in sensory cortex is gain control (Cohen and Maunsell, 2009; Reynolds and Heeger, 2009), and this mechanism extends to cross-modal contexts. Fairhall and MacAluso (2009) presented two simultaneous visual streams (speaking lips in the left and right hemifields) with a central auditory stream (spoken words) congruent with one stream and incongruent with the other. Attending to the congruent rather than the incongruent stream increased the blood-oxygen-level-dependent (BOLD) response in early retinotopic visual cortex (V1/V2), the fusiform gyrus, the superior temporal sulcus (STS), and the superior colliculus. Because the amount of multisensory information was held constant, this reflects a joint requirement for attention and cross-modal congruence rather than a response to bimodal input *per se*. A similar enhancement appears for illusory percepts. In the sound-induced flash illusion (SiFI), where a single flash with two beeps is perceived as two flashes, the extrastriate visual ERP of the illusory flash is amplified when spatial attention is directed to the stimuli (Mishra et al., 2007, 2010).

Gain modulation also operates across modalities. In humans, attending to one modality can enhance activity in the sensory cortex of another. Spatially congruent tactile stimulation enhances visual-cortical responses to visual targets via back-projections from multimodal parietal cortex (Macaluso et al., 2000). Attending to one location modulates evoked potentials to the unattended modality at the same location, producing cross-modal effects in both somatosensory and visual P200 components (Harjunen et al., 2017). Comparable gain control appears in rodents. During a visuo-tactile detection task, neurons in rat vibrissa area somatosensory cortex (vS1) showed multiplicative gain enhancement of tactile responses when touch was prioritized over vision (Lee et al., 2016), allowing attention to bias the feedforward stream from its earliest stages.

### 2.2 Temporal modulation

Beyond response amplitude, attention shapes the temporal dynamics of sensory representations. One signature is the speeding of evoked responses, the prior-entry effect. In a cross-modal temporal-order judgment task, visual-cortex ERP latencies were shorter when participants attended to vision than to touch (Vibell et al., 2007), whereas children with weaker attentional engagement showed delayed evoked-response latencies in visual, auditory, and inferior parietal cortex during multisensory trials (Pervin et al., 2025), confirming that attention modulates the timing, not only the gain, of sensory-cortex responses.

Attention also acts on ongoing cortical oscillations. While gamma-band oscillations have long been linked to visual attention through local synchronization and long-range coupling with frontal regions (Fries et al., 2001; Gregoriou et al., 2009), lower-frequency rhythms carry attentional signatures in cross-modal contexts. In monkeys performing an intermodal selection task with rhythmic audiovisual streams, delta-band oscillations in V1 entrained to the attended stream, aligning the high-excitability phase with task-relevant events to increase response gain and shorten reaction times (Lakatos et al., 2008). Cross-frequency coupling further linked the delta phase to the gamma power. Attention also reshapes the duration of sensory-cortex engagement, not only its latency and phase. Optogenetic inactivation in mice showed that the window during which V1 is causally required to detect a visual stimulus lengthens when an auditory modality must also be monitored (Oude Lohuis et al., 2022). Late-onset inactivation spared early bottom-up responses but impaired detection by interrupting a later, report-related phase of V1 activity that scaled with task demand. Together, faster latencies, entrained phase, and extended engagement yield a more temporally selective feedforward signal under cross-modal attention.

### 2.3 Cross-modal inhibition

Attentional modulation in the sensory cortex is not exclusively excitatory. Attending to one modality can suppress processing in another. Using 7T fMRI, Gau et al. (2020) showed that attending to auditory rather than visual stimuli suppressed representations of the central visual field in the human visual cortex. This aligns with Mozolic et al. (2008), who demonstrated a bidirectional effect in which attention to either audition or vision produces cross-modal deactivation in the cortex of the unattended modality, summating with stimulus-driven suppression.

A circuit-level mechanism is described in mice. Iurilli et al. (2012) found that a sound burst drives local GABAergic inhibition of supragranular pyramidal neurons in V1 via cortico-cortical projections from auditory cortex, reducing visually evoked synaptic responses and impairing detection of dim flashes. Acute pharmacological blockade of GABAergic transmission in V1 abolished both, indicating that V1 implements the suppression locally rather than inheriting it. Comparable sound-driven hyperpolarization in S1 suggests that local GABAergic circuits driven by long-range cortico-cortical input may be a general motif for cross-modal inhibition (Iurilli et al., 2012). Weiler et al. (2024) extended this motif to touch. In a sub-region of mouse V1 where visual and whisker representations overlap, contralateral whisker stimulation suppressed visually evoked activity via local fast-spiking interneurons receiving cortico-cortical input from somatosensory barrel cortex, adding a spatial specificity set by receptive-field alignment across modalities. Although neither study manipulated attention directly, the circuit they describe provides a plausible substrate for the attentional suppression of unattended sensory representations seen in the human studies above. Whether this local inhibition is causally necessary for the attentional control of multisensory perception remains to be tested.

## 3 Association cortex

The robust local modulations in sensory cortices do not arise in isolation. Association cortices, particularly the posterior parietal cortex (PPC) and STS, act as hubs where multiple sensory streams converge into a multisensory representation (Choi et al., 2023; Rohe and Noppeney, 2016; Sereno and Huang, 2014), while also receiving heavy top-down signals from frontal cortex (Behrmann et al., 2004; Corbetta and Shulman, 2002; Noudoost et al., 2010). Attentional modulation in the association cortex is different from that in the sensory cortex. It is integration-specific, emerging when the converging inputs are congruent and therefore eligible to be bound.

### 3.1 Pre-stimulus activity modulation

Association cortices exhibit attentional modulation prior to stimulus onset. Pre-stimulus beta-band power was stronger in temporal cortex before illusory perception of the SiFI (Keil et al., 2014) and the McGurk effect (Keil et al., 2012). Stronger pre-stimulus beta power over parieto-occipital scalp also preceded non-illusory judgments of audiovisual simultaneity (Yuan et al., 2016). Besides beta, lower pre-stimulus alpha power over posterior scalp accompanied higher sensitivity to audiovisual temporal lags (London et al., 2022). Anticipatory effects are not restricted to local power, as gamma-band coherence preceding the auditory stimulus predicted the auditory bias on visual perception (Hipp et al., 2011). In contrast, increased pre-stimulus activity in default-mode network regions, including the left angular gyrus, predicted slower responses (attentional lapses), most strongly in the visual modality (Su et al., 2020). Pre-stimulus activity in association cortices can thus reflect either preparatory biasing of integration or task disengagement, although the evidence remains largely correlational.

### 3.2 Congruence-dependent gain

Unlike the gain control seen in the sensory cortex, attentional enhancement in the association cortex is congruence-dependent. It emerges only when auditory and visual signals are congruent and thus candidates for integration. This condition operates over both space and time. Spatially, audiovisual congruence modulated PPC responses (Nardo et al., 2014), and attention to a congruent audiovisual stream enhanced responses in the STS, an associative integration site (Fairhall and MacAluso, 2009). Conversely, competing input across modalities can suppress spatial coding. In macaques, lateral intraparietal (LIP) neurons encoded auditory spatial location only when no central fixation light was present in the visual scene (Gifford and Cohen, 2004). Temporal correspondence has a parallel effect. The STS responds more strongly to temporally coincident than non-coincident audiovisual stimuli (Noesselt et al., 2007), a stimulus-driven sensitivity rather than an effect of directed attention *per se*. Yet top-down attention shapes these responses as well. STS activity was greater for attended, audiovisually congruent stimuli than for incongruent or unattended stimuli, indicating that STS modulation is contingent on both attention and audiovisual congruence (Morís Fernández et al., 2015).

These modulations are causally necessary, not merely correlational, as shown by perturbing the association cortex. Disrupting these regions alters multisensory binding in illusions. TMS over the right parietal cortex reduced illusory audiovisual binding in the SiFI (Kamke et al., 2012), and TMS over the STS disrupted the McGurk effect (Beauchamp et al., 2010). Disruption also changes non-illusory cross-modal processing. TMS over the right inferior parietal cortex impaired reflexive spatial orienting to visual and somatosensory targets following a somatosensory cue, implicating parietal cortex in cross-modal orienting within and between modalities (Chambers et al., 2007), whereas anodal tDCS over the right PPC sped detection of auditory, visual, and bimodal audiovisual targets (Bolognini et al., 2010).

### 3.3 Cross-modal inhibition

Attention-dependent cross-modal inhibition also occurs in association cortex, where rodent work shows that local interneuron circuits conditionally gate how sensory inputs converge. In mouse PPC, auditory-cortical inputs preferentially recruit parvalbumin-positive (PV+) interneurons that feedforward-inhibit concurrent visual inputs, producing auditory dominance during audiovisual conflict (Song et al., 2017). Muscimol inactivation of PPC or optogenetic silencing of its PV+ neurons abolished this dominance without impairing unisensory perception, while activating them enhanced it. The dominance is behaviorally flexible. Prolonged locomotion shifts decisions toward vision by inhibiting auditory-cortical neurons projecting to PPC, via secondary motor cortex projections to auditory cortex (Choi and Lee, 2025). Although locomotion is a behavioral state rather than an attentional manipulation, this circuit is a candidate motif by which top-down signals could flexibly bias cross-modal representations. Together with the human perturbation studies above, these findings establish association cortices as an integration hub for the attentional modulation of multisensory processing.

This behavioral-state dependence indicates that cross-modal weighting is not fixed. Modality-specific differences may also arise from the distinct functional roles of the senses: under a central–peripheral dichotomy, “peripheral” senses such as audition and peripheral vision primarily orient attention, whereas “central” senses such as foveal vision recognize the selected input (Zhaoping, 2023). How these functional distinctions interact with the modulations reviewed here remains untested, and multisensory integration should not be assumed to operate uniformly across modalities, eccentricities, and task demands.

## 4 Prefrontal cortex

Unlike sensory and association cortices, where attention primarily modulates stimulus-driven responses, prefrontal cortices (PFCs) show activity related to task-level operations. Extensive work shows that the prefrontal cortex exerts top-down control of attention over diverse cortical areas (Corbetta and Shulman, 2002; Kastner and Ungerleider, 2000; Noudoost et al., 2010; Zikopoulos and Barbas, 2007). We propose that prefrontal cortices exert top-down control in multisensory conditions requiring attention, selecting which modality to attend, detecting conflicts between modalities, and resolving the resulting uncertainty.

### 4.1 Modality selection

Prefrontal activity is modulated when attention shifts between sensory modalities. Whereas visual- and auditory-cortex activity depended on which modality was attended, the superior prefrontal cortex (with PPC) showed transient increases time-locked to the moment of cross-modal attentional shift (Shomstein and Yantis, 2004). During divided attention between vision and audition, sensory-cortex activity decreased relative to focused single-modality attention while left lateral PFC activity increased selectively (Loose et al., 2003). A direct comparison of within-modal and cross-modal divided attention showed that the cross-modal task produced more extended, bilateral frontoparietal activation and stronger anterior cingulate and thalamic engagement, reflecting coordination across modalities rather than within-modality demand (Vohn et al., 2007). PFC is therefore preferentially engaged when attentional resources must be coordinated across modalities and also shows a modality-specific organization. Lateral prefrontal (and superior parietal) areas are differentially recruited for auditory versus visual spatial localization (Bushara et al., 1999). During multisensory cognitive control, the lateral PFC is stratified along its rostro-caudal axis, with rostral LPFC preferentially engaged during auditory attention and caudal LPFC supporting supramodal control during audiovisual conflict (Mayer et al., 2017).

The necessity of frontal cortex for this flexible selection is established by causal manipulations in rodents. Bilateral lesions of rat medial frontal cortex selectively impaired shifting of attentional set between perceptual dimensions (e.g., odor and texture), sparing initial discrimination, reversal learning, and intradimensional shifts (Birrell and Brown, 2000). In mice, medial PFC lesions likewise impaired extradimensional set-shifting, whereas orbital PFC lesions disrupted reversal learning, a double dissociation indicating that the medial PFC contribution is dissociable from adjacent prefrontal functions (Bissonette et al., 2008). Temporally precise optogenetic perturbation of PFC diminished mice’s ability to select between conflicting visual and auditory stimuli, an effect dependent on PFC interactions with the sensory thalamus rather than sensory cortex (Wimmer et al., 2015). The downstream circuit was later identified. PFC regulates sensory thalamic gain not directly but through a basal ganglia-to-thalamus pathway, and perturbing this PFC-basal ganglia-thalamus route impaired selection between vision and audition, primarily by failing to suppress the distracting modality (Nakajima et al., 2019). Together, these findings demonstrate the necessity of frontal cortex, and of a specific top-down subcortical circuit, for cross-modal attentional flexibility.

### 4.2 Conflict monitoring and resolution of uncertainty

Prefrontal cortices, especially the anterior cingulate cortex (ACC), are well known for conflict monitoring (Botvinick et al., 2004), and such signals also arise during multisensory conflict. In an audiovisual selective-attention task, the left inferior frontal sulcus showed an evidence-accumulation profile, weighting auditory and visual inputs by their reliability to form a perceptual decision under conflict (Noppeney et al., 2010). The ACC and anterior insula were activated by incongruent audiovisual speech under attention (Morís Fernández et al., 2015). Beyond detecting incongruent stimuli, prefrontal cortex processes the uncertainty that conflict generates. The left inferior frontal sulcus combines top-down congruency expectations with bottom-up incongruency signals to arbitrate between integration and segregation (Gau and Noppeney, 2016). Perception of the McGurk illusion evokes activation from the inferior frontal sulci to the pre-SMA that is accounted for by perceptual uncertainty (Dong et al., 2024), and a separate McGurk study found ACC and inferior frontal gyrus activation stronger when the illusion was perceived than on physically identical non-illusory trials (Morís Fernández et al., 2017). The role of PFC in multisensory conflict thus extends beyond mismatch detection to the resolution of uncertainty arising from cross-modal incongruency.

## 5 Connectivity between areas

The preceding sections establish that attention modulates multisensory processing at each cortical level, but these modulations do not occur in isolation. If attention operates hierarchically, directed connectivity between regions should shift with attentional state and multisensory demand. Here, we examine state-dependent coupling across three cortical interfaces (association-sensory, frontal-association, and frontal-sensory) and then the frequency organization of these directed influences.

### 5.1 Association-sensory cortex

Several lines of evidence show attention-dependent connectivity between association and sensory cortices in cross-modal settings. In the visual stream, a spatially congruent tactile stimulus enhanced visual-cortical activity, and effective-connectivity analysis indicated that this crossmodal influence reached unimodal visual cortex via back-projections from multimodal parietal areas (Macaluso et al., 2000). Frank et al. (2020) provided causal evidence for such parietal gating. Inhibitory TMS over PPC reduced the visual-attention-induced suppression of vestibular cortex (PIVC), indicating that PPC actively gates sensory-cortex activity in favor of the attended modality. In the auditory stream, beta-band coupling between the temporal gyrus and the auditory cortex was elevated before illusory perception of the SiFI while phase synchrony with visual areas decreased (Keil et al., 2014), suggesting that oscillatory coupling may arbitrate the weighting of auditory and visual inputs. Choi and Lee (2025) proposed a circuit-specific mechanism in which auditory inputs to the parietal cortex are selectively suppressed during locomotion when competing audiovisual stimuli are present. Although this targeted behavioral state rather than directed attention, it identifies a top-down gating motif by which association-sensory connectivity can be flexibly reconfigured.

### 5.2 Frontal-association cortex

The fronto-parietal network has been characterized as the substrate for top-down attentional control, with parietal regions encoding spatial priority and frontal regions handling selection and switching (Corbetta and Shulman, 2002; Petersen and Posner, 2012). This network is recruited when cross-modal coordination is required. Shifting attention between vision and audition coactivates parietal and prefrontal cortex (Shomstein and Yantis, 2004). It subdivides into dorsal and ventral streams with complementary roles. The dorsal stream was recruited by cross-modal semantic congruence regardless of task demand, whereas the ventral stream was recruited only under high task demand (Mastroberardino et al., 2015). Beyond coactivation, beta-band coherence between mid-cingulate and superior temporal cortex was modulated by audiovisual congruence, consistent with increased fronto-association engagement during cross-modal conflict (Friese et al., 2016).

### 5.3 Frontal-sensory cortex

Frontal cortex also shows attention-dependent connectivity directly with sensory cortices, indicating that top-down control operates through both direct and indirect pathways. At the activation level, dorsolateral PFC and dorsal ACC were engaged during auditory attention in an audiovisual Stroop task and showed higher resting-state connectivity with auditory cortex (Mayer et al., 2017). At the oscillation level, frontal cortex exerted control through increased low-frequency phase synchronization with sensory cortex for attended audiovisual stimuli (Friese et al., 2016). At the circuit level, primate recordings showed that attention selects between auditory and visual streams by resetting the phase of ongoing oscillations in sensory cortex (Lakatos et al., 2008). This is widely interpreted as frontal top-down control, though the frontal origin was not directly demonstrated. Frontal cortex thus exerts cross-modal attentional control over sensory representations through both correlated activation and active phase coupling.

### 5.4 Feedforward and feedback coherence

Directed influences between cortical areas are organized by frequency band. In the visual system, feedforward influences are carried predominantly by gamma- and theta-band synchronization and feedback influences by alpha/beta-band synchronization, in macaque (Bastos et al., 2015) and in humans (Michalareas et al., 2016). These observations are consistent with the communication-through-coherence principle, in which coherence between two regions gates their effective connectivity (Fries, 2005). This organization accounts for the band-specific effects described in the preceding subsections. Elevated beta-band coupling between association and sensory cortex, and between frontal and association cortex, during illusory binding and cross-modal conflict (Keil et al., 2014; Friese et al., 2016) is consistent with feedback signaling. Low-frequency frontal-to-sensory phase synchronization and phase reset during attended stimuli (Friese et al., 2016; Lakatos et al., 2008) are consistent with top-down entrainment of sensory cortex. Applied to cross-modal attention, this framework predicts that top-down gating of an unattended modality appears as alpha/beta-band coupling from frontal and association cortex to sensory cortex, while the attended stream is propagated forward to association and frontal cortex in the gamma band. Cross-frequency coupling, in which top-down beta enhances bottom-up gamma (Richter et al., 2017), is a candidate mechanism by which selection at higher levels is translated into gain control at the sensory level.

## 6 Conclusion

Across the cortical hierarchy, attention acts not as a uniform amplifier but as a set of level-specific operations during multisensory perception. Sensory cortices adjust local gain and temporal precision, association cortices bias how converging inputs are integrated, and the prefrontal cortex selects the relevant modality, monitors conflict, and resolves the resulting uncertainty. A common motif recurs at each level (the attended modality is enhanced while the competing one is suppressed), and these complementary effects are bound together by attention-dependent inter-areal recurrent connectivity and state-dependent coupling. This division of labor draws convergent correlational and causal support from human, primate, and rodent studies.

This framework extends existing models of attentional priority to multisensory conditions. Normalization (Reynolds and Heeger, 2009), biased-competition (Desimone and Duncan, 1995), and priority-map accounts (Bisley and Goldberg, 2010) were developed largely for unisensory vision, and recent efforts to consolidate the sources of priority into a single mechanism, whether across evolutionary, learned, and task-driven timescales (Saghravanian, 2025) or under a single memory-dependent attentional control state (Anderson, 2024), operate within a predominantly single-modality frame. These models specify how goals, salience, and history are combined, but not how priority is computed when signals must be integrated across the senses. The framework advanced here addresses this cross-modal, multi-level dimension and predicts that a congruence-dependent cross-modal priority signal emerges in association cortex, and that disrupting this signal selectively impairs integration without abolishing unisensory priority, consistent with the causal evidence reviewed above (Beauchamp et al., 2010; Kamke et al., 2012; Song et al., 2017).

Attention is therefore better characterized as an integration-sensitive process than as a uniform amplification of sensory signals, in that selection and gain are conditioned on whether cross-modal signals are integrable. Two questions remain open. The direction of influence between levels is largely inferred rather than measured, and several of the inhibitory circuits identified in rodents were engaged by behavioral state rather than by directed attention, leaving it unclear whether attention recruits the same machinery. Resolving both will be central to a mechanistic understanding of attention spanning the cortical hierarchy.
